# B cell subset distribution in human bone marrow is stable and similar in left and right femur: An instructive case

**DOI:** 10.1371/journal.pone.0212525

**Published:** 2019-02-22

**Authors:** Annika Wiedemann, Andreia C. Lino, Thomas Dörner

**Affiliations:** 1 Charité Universitätsmedizin Berlin, Department of Medicine/Rheumatology and Clinical Immunology, Berlin, Germany; 2 German Rheumatism Research Center Berlin (DRFZ), Berlin, Germany; Institut Cochin, FRANCE

## Abstract

The bone marrow (BM) is, in addition to being the site of B cell development, a tissue that harbors long-lived plasma cells (PC), the cells that protect the body against foreign antigens by continuous production of antibodies. Nothing is known about the long-term stability and functionality of both B cells and PC in the BM at the individual donor level since repeated sampling possibilities outside of oncology are scarce. Here, we had the opportunity to obtain BM samples from a patient undergoing bilateral total hip arthroplasty half a year apart. We observed that the frequencies of the analyzed B cell and PC subsets were similar despite a time of six months in between and sampling on left and right side of the body. Additionally, B cell receptor stimulation led to comparable results. Our data suggest that composition and functionality of B cells are stable in the BM of adults at the individual donor level.

## Introduction

The bone marrow (BM) is the primary site of hematopoiesis and B cell development. In addition, it enables survival of long-lived plasma cells (PC) as ultimately differentiated B cells. BM microenvironments play essential roles in supporting these processes, and niches providing survival factors are crucial for the maintenance of both hematopoietic progenitors and long-lived PC. Stromal cells, megakaryocytes and eosinophils were shown to provide soluble factors like interleukin (IL)-6, IL-7, a proliferation-inducing ligand (APRIL), integrin alpha4 and ligands for chemokine receptors, such as CXCL12, to support the survival of the aforementioned cell types [1–5]. Among human BM PC, a subset lacking the expression of CD19 considered to be long-lived was found to carry a pro-survival and more mature phenotype than their CD19^+^ counterparts, whereas only low frequencies of CD19^-^ PC can be found in tonsils, spleen and peripheral blood [6–8]. In a more recent work, long-lived CD19^-^ PC were also identified in the human intestine [9]. A detailed analysis of human tetanus toxoid (TT)-specific CD27^+^CD20^+^ memory B cells (mBC) in different tissues and in the periphery showed that the phenotype of mBC does not differ in the tissues suggesting that mBC patrol through the entire body rather than carrying a tissue-specific phenotype [10]. It has been shown that B cell subsets in the peripheral blood are stable over months [11] but notably, nothing is known about the longitudinal stability and comparability of such subsets in the BM within a healthy individual. To obtain primary tissue from patients and healthy donors is challenging and the availability largely depends on remaining surgical materials, which makes it highly unlikely to receive tissue from the same individual during follow-up. Data on human BM outside oncology are scarce but of substantial relevance while the availability of material is limited. Thus, it had not been possible to assess the distribution of B cell subsets and PC within a tissue like BM longitudinally in healthy individuals. A recent study in patients with acute lymphoblastic leukemia assessed the presence of leukemic clones in paired BM samples and could show that 86% of the subclones were present in both samples at the same time. Furthermore, 83% of the clones were found in paired BM and peripheral blood samples [12].

Here, for the first time, we had the unique opportunity to analyze two bone marrow samples of a 52-year-old woman who underwent bilateral total hip arthroplasty due to coxarthrosis half year apart. After a first sample from the left femur, subsequently a second sample from the right femur could be analyzed. All experiments were carried out blinded since we only learned after final data analysis that the second sample came from the same person.

## Material and methods

### Donor

Bone marrow samples were obtained from an individual (female, 52 years old) suffering from coxarthrosis undergoing bilateral total hip arthroplasty six months apart. Except for hypothyroidism and intake of L-thyroxin, no inflammation or immune-related manifestation was recorded. The study was approved by the local ethics committee of Charité Universitätsmedizin Berlin and written consent was obtained from the patient.

### Isolation of mononuclear cells

Mononuclear cells from the bone marrow were isolated as previously described [6]. Briefly, samples were fragmented, rinsed with PBS (Biochrom, Berlin, Germany) and filtered with a 70 μm cell strainer (BD Falcon, Heidelberg, Germany). The obtained cell suspension was used for a density gradient centrifugation with Ficoll Paque (GE Healthcare, Buckinghamshire, UK). The collected mononuclear cells were washed twice with PBS and resuspended in RPMI 1640 (Thermo Fisher Scientific, Waltham, USA).

### Stainings for flow cytometry

For surface stainings, 2x10^6^ MNCs were stained for 15 min at 4°C with different combinations of antibodies. For intracellular stainings, cells were stained for 1 hour at room temperature with the respective antibodies. Anti-human antibodies (clone, manufacturer) binding to the following molecules have been used: Pacific Blue (PacB)-conjugated CD3 (UCHT1, BD Biosciences), PacB-conjugated CD14 (M52E, BD Biosciences), Phycoerithrin-Cyanin 7 (PE-Cy7)-conjugated CD19 (HIB19, Thermo Fisher Scientific), Allophycocyanin (APC)-H7-conjugated CD19 (SJ25C1, BD Biosciences) or APC-Cyanin7 (APC-Cy7)-conjugated CD19 (SJ25C1, Biolegend), Brilliant Violet (BV) 510-conjugated CD20 (2H7, Biolegend, San Diego, USA), APC-conjugated CD27 (L128, BD Biosciences), APC-Cy7-conjugated CD38 (HIT2, Biolegend) or PE-conjugated CD38 (HIT2, BD Biosciences), PE-conjugated pSYK Y^352^ (17A/P-ZAP70, BD Biosciences), Fluorescein isothiocyanate (FITC)-conjugated IgA (M24A, Thermo Fisher Scientific), PE-Cy7-conjugated IgG (G18-145, BD Biosciences), PerCpCy5.5-conjugated IgM (G20-127, BD Biosciences). For identification of dead cells, DAPI (Molecular Probes, Eugene, USA) was added to surface stained cells. Cells were analyzed with a FACS Canto II flow cytometer (BD Biosciences).

### B cell receptor stimulation

For B cell receptor (BCR) stimulation, 2x10^6^ MNCs were equilibrated with RPMI 1640 at 37°C for 30 minutes and stimulated with 30 μg/ml anti-IgM/IgG/IgA (Jackson ImmunoResearch, Ely, UK) for 5 min. To assess baseline phosphorylation, cells were treated with RPMI for 5 min (control). After stimulation, cells were lysed with Lyse/Fix Buffer (BD Biosciences), permeabilized with Perm Buffer II (BD Biosciences) according to the manufacturer’s protocol, washed with PBS/BSA/EDTA and stained intracellularly.

### Data and statistical analysis

Flow-cytometric data was analyzed with FlowJo version 7.6.5 (FlowJo, LLC). Data analysis was performed with GraphPad Prism 6 software (Graphpad Software, Inc).

## Results

**Fig 1:**
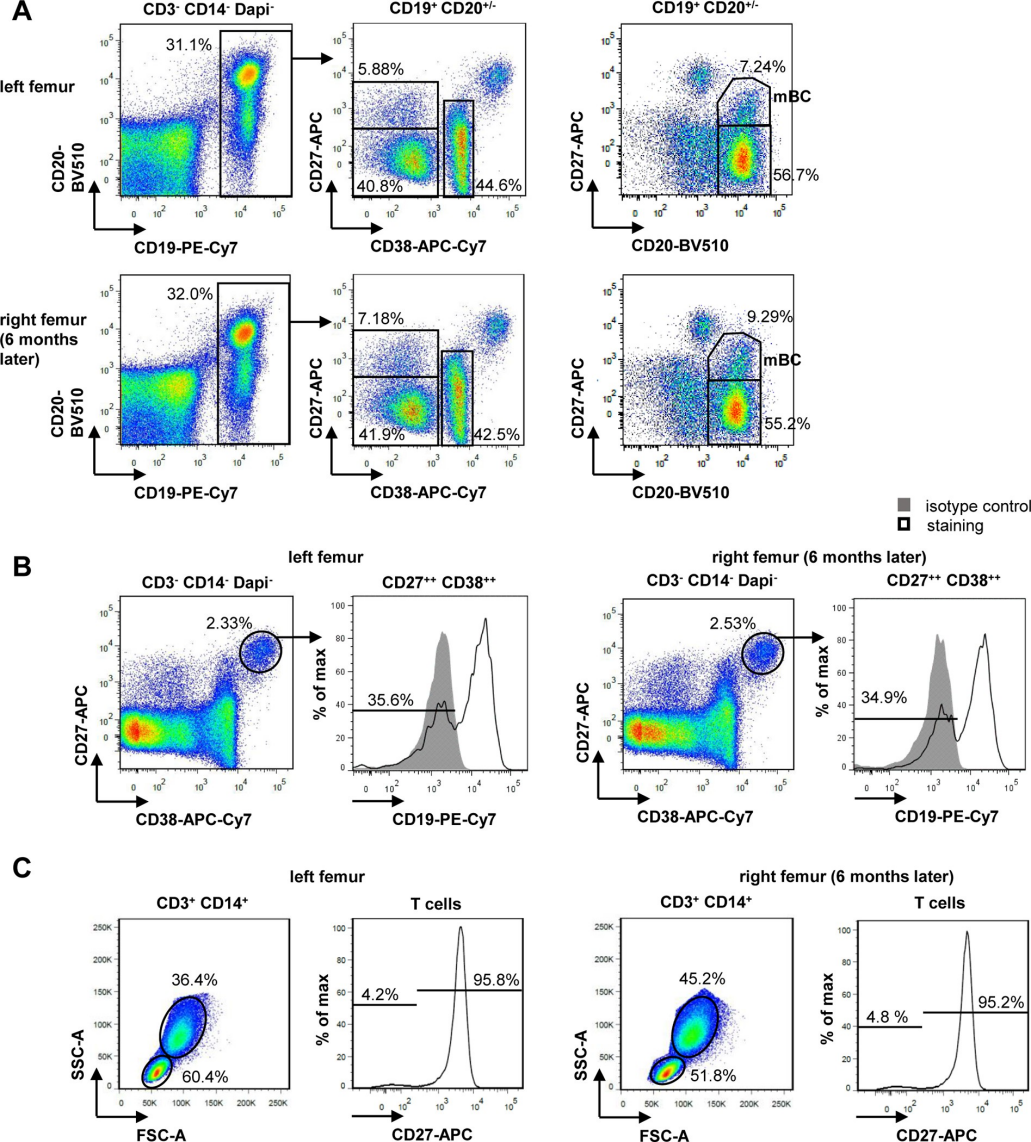
Frequencies of cells populations in the two BM samples are comparable. **A**) Dotplots of CD3^-^CD14^-^Dapi^-^ single lymphocytes to identify CD19^+^CD20^+/-^ B cells and subsequent discrimination of B cell subpopulations by either CD27/CD38 or CD27/CD20. **B**) Identification of CD27^++^CD38^++^ PC among CD3^-^CD14^-^Dapi^-^ BM MNCs and subsequent gating of CD19^-^ PC. **C**) Discrimination of T cells and monocytes among CD3^+^CD14^+^Dapi^-^ cells by their scatter properties and frequency of CD27^+^ T cells.

First, surface marker expression to identify B cells, PC, T cells and monocytes was analyzed by flow-cytometry (Fig 1). B cells and PC were identified among BM mononuclear cells (MNCs) as CD3^-^CD14^-^Dapi^-^CD19^+^CD20^+/-^ (B cells) and CD3^-^CD14^-^Dapi^-^CD27^++^CD38^++^ (PC) single lymphocytes from left and right femur specimen. T cells and monocytes were gated among CD3^+^CD14^+^Dapi^-^ cells by their scatter properties.

Analyses of the frequencies of B cells and subpopulations either by combination of CD27 and CD38 or CD27 and CD20 revealed that these were highly similar between both samples (Fig 1A). In detail, among CD19^+^CD20^+/-^ B cells, 5.88% (left femur) and 7.18% (right femur) were CD27^+^CD38^-^ memory B cells, 40.8% (left femur) and 41.9% (right femur) were CD27^-^CD38^-^ naïve B cells and 44.6% (left femur) and 42.5% (right femur) were identified as CD27^-^CD38^+^ expressing immature and transitional B cells. In a recent report the B cell phenotypes in the BM have been assessed. Here, immature CD38^+^ B cells are described to represent one third of the B cells in the BM [13]. Adding other CD38-expressing cells such as transitional B cells provides comparable percentages as seen for CD27^-^CD38^+^ B cells in our samples (42–44%). In the report, CD38^-^ B cells divide into CD27- and CD27^+^ B cells with the CD27^+^ being the less frequent subset [13]. Gating the two samples for CD27^+^CD20^+^ conventional memory B cells showed frequencies of 7.24% (left femur) and 9.29% (right femur), respectively. In the same plot, gating for conventional CD27^-^CD20^+^ naïve B cells revealed frequencies of 56.7% (left femur) and 55.2% (right femur).

Comparable results were also found for the frequency of BM PC and the percentage of CD19^-^ PC subsets in the two samples (Fig 1B). While the reported frequency of CD19^-^ BM PCs in healthy individuals ranges from 9–56% with a median of 33% [6], we found remarkably similar frequencies of 35.6% and 34.9% for the two samples (Fig 1B). We reported CD19^-^ BM PCs to have a more mature/ long lived phenotype [6], therefore these similar frequencies suggest that long-lived PC are stable in the BM. The frequency of CD27^++^CD38^++^ PC among CD3^-^CD14^-^Dapi^-^ cells was 2.33% and 2.53%, respectively. T cells comprised 60.4% and 51.8% in the two samples and monocytes 36.4% and 45.2%. The percentage of CD27^+^ cells among T cells was also highly similar, they comprised 95.8% and 95.2%, respectively (Fig 1C).

**Fig 2:**
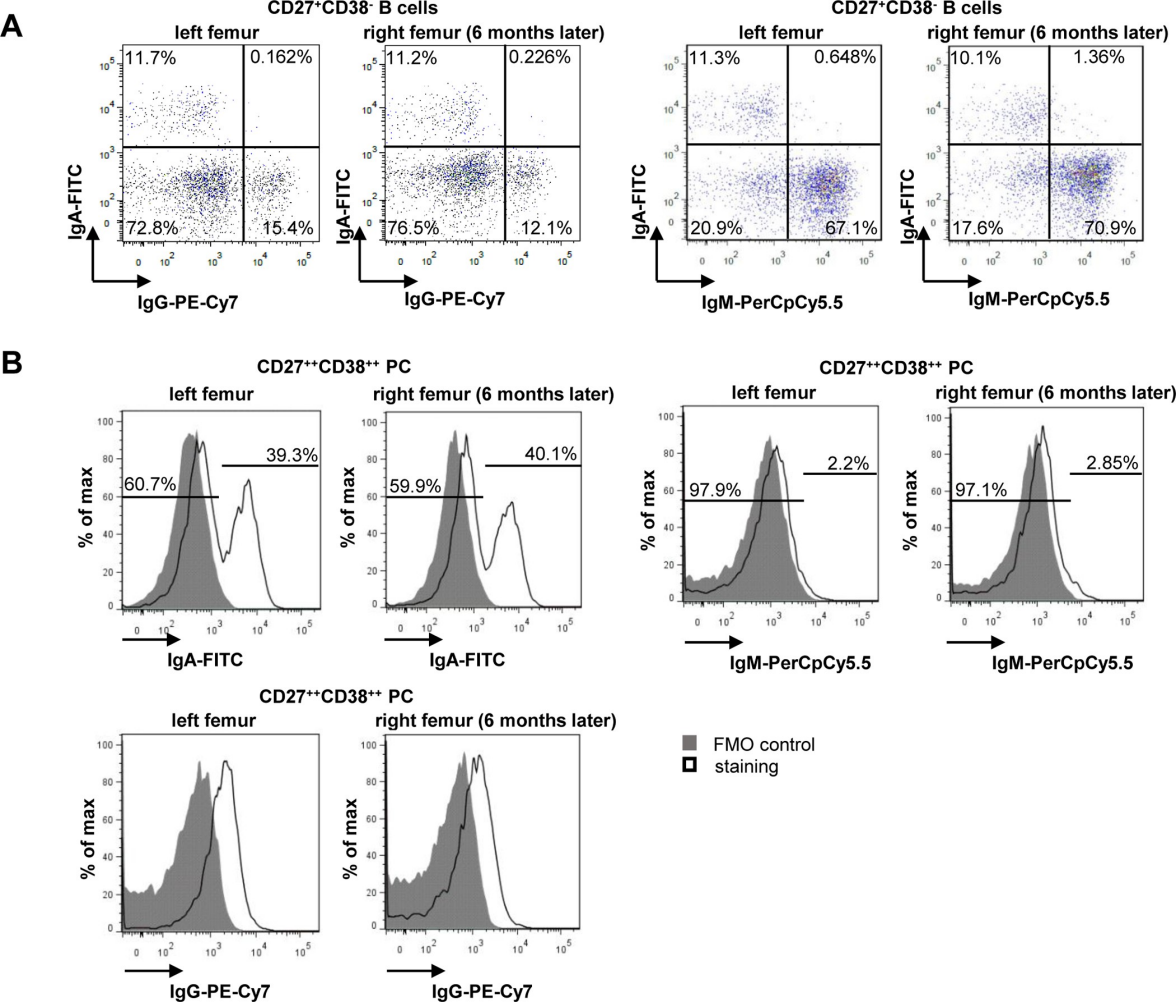
Similar immunoglobulin (Ig) expression on PC and B cell subsets. **A**) Surface staining of IgA, IgM and IgG on CD20^+^CD27^+^CD38^-^ memory B cells. **B**) Surface staining of IgA, IgM and IgG on CD27^++^CD38^++^ PC. FMO = Fluorescence Minus One.

Analysis of surface immunoglobulin (Ig) surface expression by flow-cytometry showed the same isotype distribution of IgA, IgM and IgG for PC and B cell subsets as exemplarily shown for CD27^+^CD38^-^ memory B cells (Fig 2A) and CD27^++^CD38^++^PC (Fig 2B). Here, 11.7% (left femur) and 11.2% (right femur) of CD27^+^CD38^-^ B cells expressed IgA and 15.4% (left femur) and 12.1% (right femur) expressed IgG (Fig 2A). 67.1% (left femur) and 70.9% (right femur) of CD27^+^CD38^-^ B cells expressed IgM. Among CD27^++^CD38^++^ PC, 39.3% (left femur) and 40.1% (right femur) stained positive for surface IgA (Fig 2B). Only 2.2% and 2.85% of BM PC showed a surface staining of IgM. IgG was found not to be expressed on the surface of PC as it was described before [14].

**Fig 3:**
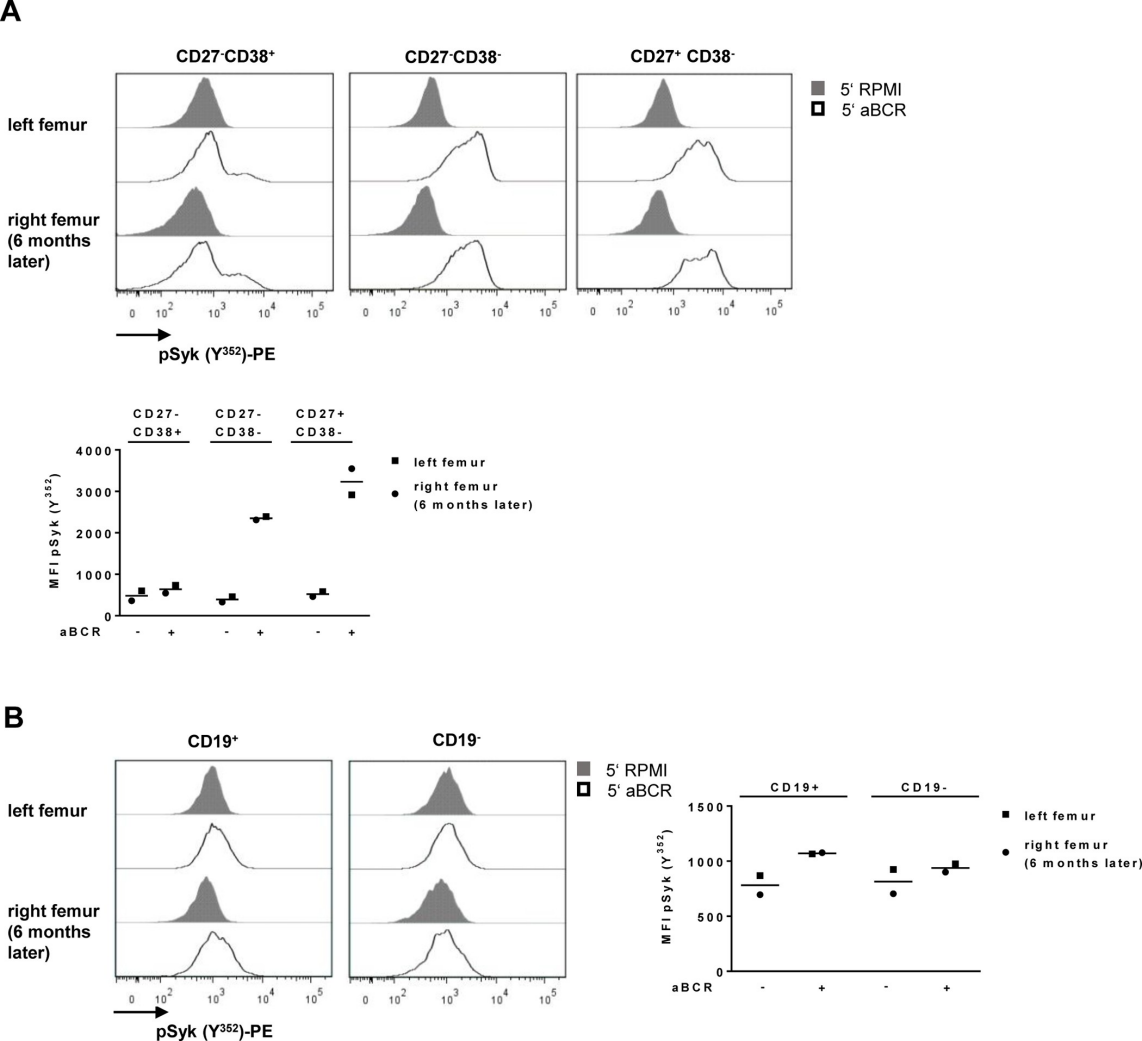
Response to BCR stimulation by BM B cell subsets is stable.BM MNCs were stimulated with 30 μg/ml anti-B cell receptor (aBCR) IgM/IgA/IgG or RPMI as control and the median fluorescence intensity (MFI) of pSykY^352^ has been assessed in both samples. **A**) Histograms and MFI of pSykY^352^ in BM B cell subsets identified by CD27 and CD38. **B**) Histograms and MFI of pSykY^352^ in CD19^+^ and CD19^-^ BM PC.

Functional characteristics of both samples were assessed after BCR stimulation with anti-BCR F(ab)_2_ IgM/IgA/IgG, lysis/fixation, permeabilization and subsequent intracellular staining of phosphorylated spleen tyrosine kinase at tyrosine Y^352^ (pSyk Y^352^). Syk is one of the proximal downstream targets of the BCR signaling cascade, which is recruited by CD79a/b ITAM and is phosphorylated by Lyn after BCR engagement. This activates downstream targets and leads to activation and differentiation of B cells [15, 16]. Here, BCR stimulation led to similar median fluorescence intensities (MFI) for the three B cell subsets discriminated by CD27 and CD38 (Fig 3A). In CD27^-^CD38^+^ B cells, which rarely express a BCR, a small increase in the MFI of pSyk Y^352^ from 598 (unstimulated control, left femur) to 734 (BCR stimulation, left femur) and 362 to 545 (right femur) was observed. CD27^-^CD38^-^ B cells showed a comparable increase of pSyk MFI in both samples from 457 to 2392 and 327 to 2312, respectively. The most prominent effect in terms of Syk phosphorylation after BCR stimulation was found in CD27^+^CD38^-^ B cells. Here, an increase of pSyk MFI from 580 to 2914 and 461 to 3548 was measured. BCR stimulation of PC led to an increase of Syk phosphorylation at tyrosine 352 as well but with a smaller magnitude than seen for CD27^-^ and CD27^+^ B cells. In CD19^+^ PC, which showed a slightly higher response to BCR stimulation, the MFI of pSyk Y^352^ increased from 869 to 1065 (left femur) and 695 to 1077 (right femur). CD19^-^ PC showed an increase of pSyk MFI from 924 to 975 and 704 to 901 (Fig 3B).

## Discussion

Data comparing frequencies of cell subsets longitudinally in the readily accessible peripheral blood is available and shows that B cell subsets in individuals can be stable for more than 70 days [11]. The BM is a place of long-term maintenance for the cells of the humoral immune system and stem cells to consistently replenish circulating cells. Thus, it should provide stability to protect the host against infections but should also be dynamic to adapt to newly faced challenges. There are changes occurring in the BM from childhood to adulthood and to senescence, as for example a change from mostly red BM to yellow BM. In adults, only some bones are left containing red BM and thus harbor immune cells. Here, we had the unique opportunity to analyze BM samples of the left and right femur of the same person who underwent bilateral total hip arthroplasty half a year apart. Since the time between the sampling is relatively short compared to a lifetime, we hypothesized the samples to be comparable although this was never shown before in healthy individuals. We did not have the opportunity to compare samples at two time points from the same sampling location and thus can only assume that the distribution at one time is the same in both femurs. This hypothesis is in line with the finding of the same leukemic clones in paired BM samples from patients at the same time [12]. On the individual donor level, our data suggest stable frequencies and comparable function of human BM PC and B cell subsets over time (at least 6 months) and a similar B lineage cell constitution between left and right femur in a healthy 52 year old female.
